# Distance Effects of Phenylpiperazine-Containing Methacrylic Polymers on Optical and Structural Properties

**DOI:** 10.1021/acs.jpcb.1c05654

**Published:** 2021-09-09

**Authors:** Beata Derkowska-Zielinska, Anna Kaczmarek-Kedziera, Malgorzata Sypniewska, Dariusz Chomicki, Robert Szczesny, Lukasz Skowronski, Viviana Figà, Oksana Krupka

**Affiliations:** † Institute of Physics, Faculty of Physics, Astronomy and Informatics, Nicolaus Copernicus University in Torun, Grudziadzka 5, 87-100 Torun, Poland; ‡ Faculty of Chemistry, Nicolaus Copernicus University in Torun, Gagarina 7, 87-100 Torun, Poland; § Institute of Mathematics and Physics, UTP University of Science and Technology, S. Kaliskiego 7, 85-796 Bydgoszcz, Poland; ∥ Euro-Mediterranean Institute of Science and Technology Palermo, via Michele Miraglia 20, 90100 Palermo, Italy; ⊥ Taras Shevchenko National University of Kyiv, 64/13 Volodymyrska Street, 01601 Kyiv, Ukraine

## Abstract

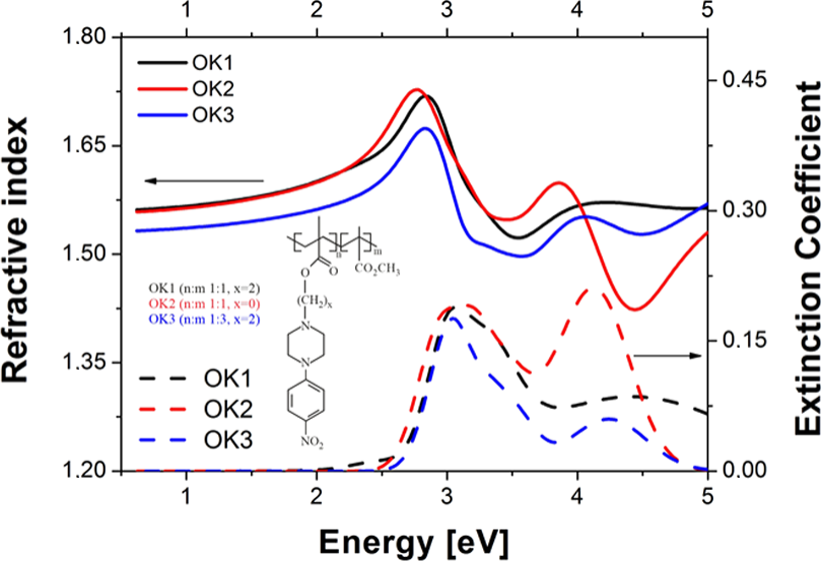

New materials based on methacrylic polymers modified with 1-(4-nitrophenyl)piperazine side chains, differing in the distance of the chromophore from the polymer main chain and/or the separation between the chromophoric units in the chain, are obtained and characterized in terms of their potential applications in optoelectronic devices. The surface, structural, and optical properties of the investigated materials are determined using atomic force microscopy, spectroscopic ellipsometry combined with transmission measurements, Raman and Fourier transform infrared spectroscopy, as well as cyclic voltammetry. The relevant model systems are additionally analyzed with quantum chemical density functional theory calculations in order to enable the generalization of the structure–photophysical property relationships for the optimization of the material features. It is found that the structural modification of the material, relying on the transit of the piperazine moiety away from the main polymer chain, leads to the hypsochromic shift of the absorption spectrum. Moreover, the lowest refractive index values are obtained for the polymer with a distant ethylene group in the side-chains and increased separation between the piperazine units. It was shown that the optical energy band gaps of the investigated piperazine-containing polymers are in the range from 2.73 to 2.81 eV, which reveals their promising potential for the advances in photovoltaics, field effect transistors, or electrochromic devices as an alternative for other widely applied polymer materials.

## 1 Introduction

In recent years, organic optoelectronic materials have been receiving much scientific attention due to their low production costs and possible rational design and precise tailoring of their properties through modifications of their chemical structure.^1^ Among them, an important class of materials are donor–π-conjugated bridge–acceptor (D–π–A) systems, which are attractive for applications such as nonlinear optical devices, organic light-emitting diodes, and organic solar cells (OSCs) because of the intermolecular charge transfer character of their excited state.^2^ Moreover, the molecular properties of these “push–pull” systems depend on the ability of the donor groups to provide electrons and acceptor groups to withdraw them. Their mutual influence on the system properties strongly depends on the character and relative positions of the substituents in the system. Modification of each of the donor, π-conjugated bridge, and/or acceptor groups is an effective way to refine the properties of these materials.^2−4^

In polymeric materials, the chromophores can be dispersed into a polymer matrix (guest–host system) or covalently bonded to a polymer side chain or main chain. Guest chromophores may deteriorate the materials properties by introducing a plasticizing effect that lowers the glass-transition temperature of the polymer and can lead to the phase separation at high chromophore loading. Therefore, despite the ease of synthesis and characterization, dispersion of chromophores in polymer matrices is considered inappropriate for numerous applications. These problems may be prevented by attaching the chromophores to a polymer chain.^5^ One of the most commonly used polymers in polymeric hybrid materials is poly(methyl methacrylate) (PMMA). It is attractive due to its hardness and colorlessness. PMMA exhibits low optical absorption and high transmittance, good insulating properties, and chemical resistance. Its glass-transition temperature ranges broadly from approximately 87 to 157 °C.^6^ Due to these properties, PMMA is also used for the preparation of the transparent gel electrolyte in solid-state electrochromic devices.^7^

Piperazine derivatives comprise a broad class of chemical compounds applied in many therapeutic areas such as antibacterial, antifungal, antimicrobial, anticancer, antitumor, and anti-inflammatory agents among others.^8−10^ They have also been widely used in chemistry as chelating or bridging ligands^11,12^ and can be introduced as a spacer group to disrupt the through-bond electronic communication between donor and acceptor units.^13^ Piperazine can also be used as a molecular dopant in solar cell systems, which can improve the efficiency and stability of the polymer solar cells.^14−16^

Piperazine consists of a six-membered saturated ring containing two nitrogen atoms at positions 1 and 4 in the ring.^8^ It is an electron-rich group, which can act as an electron-donating moiety.^11,17,18^ The presence of a nitrogen atom, bearing a free electron pair, enables n–π* transitions in addition to π–π* transitions.^4^ The proper substitution of the piperazine ring allows for obtaining attractive nonlinear materials characterized by the large hyperpolarizability values.^19^ It has also been proven recently that the substitution of the π-electron-conjugated (aromatic) system with one or more piperazine groups leads to significant bathochromic shift of the absorption bands, and their intensity is highly dependent on the protonation of the system.^20^ Therefore, the idea of the modification of the polymer chain with substituted piperazine side groups arises as an interesting way of achieving new materials with extraordinary features.

The aim of the present study is to design, synthesize, and investigate the photo-physical properties of three new methacrylic side-chain polymers based on 1-(4-nitrophenyl)piperazine for their potential applications in OSC devices. The proposed systems differ in the distance of a chromophore from the main polymer chain and the separation between two adjacent chromophore units in the chain. The influence of these modifications on the material surface and structural and optical properties is analyzed in order to establish the structure–optical property relationship for this class of piperazine materials. Since, in general, these factors can have only a minor effect, they act synergistically, enhancing the material features, or cancel each other, and the process of the material design becomes multidimensional; this thorough study is supported by the theoretical density functional theory (DFT) calculations, demonstrating the influence of the intramolecular interactions between the chromophore units in the system on its properties. Additionally, cyclic voltammetry (CV) measurements have been performed in order to determine the energy band gap, electron affinities, and work functions for the obtained materials.^21^ The knowledge of the optical energy band gap provides valuable information on the choice of materials for the production of optoelectronic devices.

To the best of our knowledge, the synthesis and modification of piperazine-substituted methacrylic materials for the optimization of their optical properties are presented for the first time. Our measurements show that the studied piperazine derivative polymers, thanks to their unique optical properties combined with low energy band gaps and easy chemical modification, are excellent materials for future applications in miniaturized optoelectronic systems such as photovoltaic devices, field effect transistors, or electrochromic devices.^22^ Further on, the results of the present study may be employed for the rational design of the new polymer materials for the practical exploitation of sustainable energy sources or in artificial lightning. One need to underline that the present study is the basic research and should be treated as the first step toward novel optoelectronic materials. The analysis of the influence of the structural and electronic factors such as the electron-donating or electron-withdrawing groups’ presence, the π-electron delocalization, and the way of introduction of the considered chromophores into the polymer chain on the optical properties are perceived as the mandatory relations, which need to be determined before further optoelectronic research, including the precise ionization potential (IP), electron affinity, current density, open circuit voltage, shunt resistance, fill factor, and power efficiency.

## 2 Methods

### 2.1 Materials

#### 2.1.1 2-[4-(4-Nitrophenyl)piperazin-1-yl]ethanol

A solution of bromoethanol (3.1 mmol), 1-(4-nitrophenyl)piperazine (3 mmol), potassium carbonate (12 mmol), and acetone (30 mL) was heated at 80 °C for 20 h. The cooled reaction solution was filtered to remove inorganic materials and then evaporated down. The crude product was purified using column chromatography on silica gel with ethyl acetate, to give a yellow-brown oil; yield, 70%.

#### 2.1.2 Synthesis of Arylpiperazinyl Methacrylates: General Procedure

0.005 mol methacryloyl chloride was added dropwise to a mixture of 0.005 mol triethylamine and 0.0027 mol 1-(4-nitrophenyl)piperazinyl alcohol in 20 mL THF. After being stirred for 12 h at 0 °C, the reaction mixture was treated with dichloromethane, and the organic layer was washed with an aqueous solution of NaCl, followed by drying over magnesium sulfate and the removal of the solvent to give a solid. The crude product was recrystallized from ethanol to afford yellow powdery crystals.

#### 2.1.3 (2-Methylacryloyl)-4-(4-nitrophenyl)piperazine

^1^H NMR (400 MHz, CDCl_3_): δ 8.14–8.10 (m, 2H), 6.80 (m, 2H), 5.60 (s, 1H, ethylene), 6.15 (s, 1H, ethylene), 3.40 (t, 2H), 2.70–2.64 (m, 6H), 1.96 (s, 3H).

#### 2.1.4 2-[4-(4-Nitrophenyl)piperazin-1-yl]ethyl 2-Methylprop-2-enoate

^1^H NMR (400 MHz, CDCl_3_): δ 8.15–8.11 (m, 2H), 6.85–6.80 (m, 2H), 5.60 (s, 1H, ethylene), 6.15 (s, 1H, ethylene), 4.25 (t, *J* = 5.8 Hz, 2H), 3.44 (t, *J* = 5.2 Hz, 4H), 2.72–2.65 (m, 6H), 1.96 (s, 3H).

In the case of polymer OK1, in anhydrous *N*,*N*-dimethylformamide (2.5 mL) was dissolved 2-[4-(4-nitrophenyl)piperazin-1-yl]ethyl 2-methylprop-2-enoate (0.19 g, 0.59 mmol), methylmethacrylate (0.059 g, 0.59 mmol), and 2,2-azobisisobutyronitrile (0.0024 g, 0.015 mmol). The mixture was degassed and then heated at 80 °C for 24 h. The solution was poured into methanol to precipitate the polymer. The precipitation was repeated from *N*,*N*-dimethylformamide to methanol to give OK1 (70%). *T*_g_ is 109 °C; *M*_w_ is 33 kDa; and *M*_w_/*M*_n_ is 1.6. Polymer OK2 was synthesized in the same manner as OK1 to give purified OK2 (65%). *T*_g_ is 148 °C; *M*_w_ is 32 kDa; and *M*_w_/*M*_n_ is 1.7. Polymer OK3 was synthesized as presented above with a 3/1 molar ratio of methyl methacrylate (MMA) and 2-[4-(4-nitrophenyl)piperazin-1-yl]ethyl 2-methylprop-2-enoate in the initial mixture. Yield, 72%. *T*_g_ is 112 °C; *M*_w_ is 35 kDa; and *M*_w_/*M*_n_ is 1.85.

The copolymer ratio was determined by the integration of relevant ^1^H NMR signals. Their structures were confirmed by ^1^H NMR spectra, as well as the respective composition in both monomers (n/m ratio).

#### 2.1.5 Poly[MMA-*co*-[4-(4-nitrophenyl)piperazin-1-yl]ethyl2-methylprop-2-enoate] OK1/OK3

^1^H NMR (400 MHz, CDCl_3_): δ 2–0.5 (CH_3_ backbone), 2.72–2.65 (6H), 3.33 (2H), 3.58–3.4 (OCH_3_), 4.4–4.1 (4H), 6.81 (2H), 8.15 (2H).

#### 2.1.6 Poly[MMA-*co*-[4(2-methylacryloyl)-4-(4-nitrophenyl)piperazine] OK2

^1^H NMR (400 MHz, CDCl_3_): δ 1.5–0.5 (CH_3_ backbone), 2.72–2.65 (6H), 3.33 (2H), 3.58–3.4 (OCH_3_), 6.80 (2H), 8.13 (2H).

**Figure 1:**
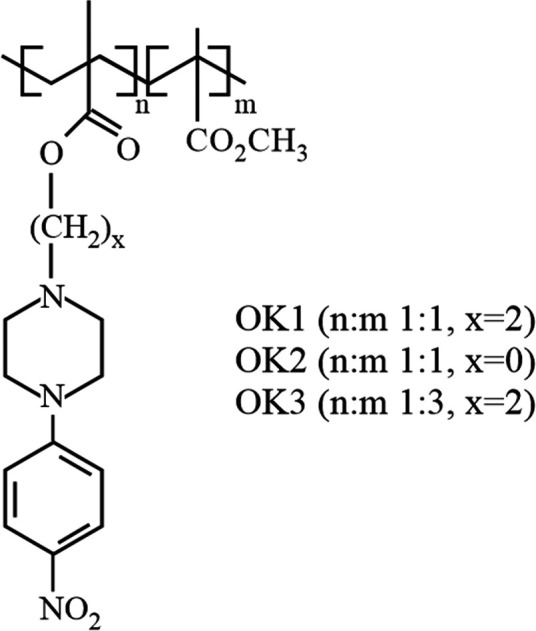
Structures of synthesized methacrylic polymers based on 1-(4-nitrophenyl)piperazine.

Figure 1 shows the molecular structures of methacrylic polymers based on 1-(4-nitrophenyl)piperazine. It can be seen that these compounds differ only in the length of the side chain and copolymer ratios in final polymers.

#### 2.1.7 Thin-Film Preparation

Homogeneous solutions of methacrylic polymers with a nitrophenylpiperazine moiety in dichloroethane (60 g/L) were filtered through 0.4 μm membranes and cast by spin-coating onto glass substrates for 60 s at 1000 rpm. The residual solvent was removed from films by heating them for 3 h at 70 °C.

### 2.2 Measurements

The ^1^H NMR (400 MHz) spectra were recorded on a Mercury (Varian) 400 spectrometer. Chemical shifts are in ppm with reference to the internal standard tetramethylsilane.

A Q20 model of differential scanning calorimetry (TA Instruments) with a continuous N2 purge was used to determine the glass and phase transition temperatures (*T*_g_) of all polymers. Two scans were run at a heating rate of 10 °C/min up to 200 °C, followed by a cooling to 20 °C, giving the values of *T*_g_.

The molecular weights of polymers were determined by gel permeation chromatography (GPC) using a chromatograph equipped with Spectra SYSTEM RI-150 and Spectra SYSTEM UV2000 detectors. THF was used as an eluent at a flow rate of 1 mL/min at 35 °C. Polystyrene standards (580–4.83 × 103 g/mol) were used for calibration.

Transmission spectra were recorded on a Cary 5000 spectrophotometer (Agilent) in the range from 0.5 to 5.0 eV at room temperature.

Ellipsometric azimuths (Ψ and Δ) were measured using the V-VASE device (J.A. Woollam Co., Inc.) for three angles of incidence (65, 70, and 75°). The measurements were performed in the spectral range 0.5–5.0 eV at room temperature. The optical constants (refractive index and extinction coefficient) and the thickness of methacrylic polymers based on 1-(4-nitrophenyl)piperazine thin films were determined using a four-medium optical model of the sample (i.e. glass/polymer layer/rough layer/ambient). The optical constants of the studied polymer films were parameterized using the Gauss-shape and Sellmeier-type dispersion relations in the absorption and transparent regimes, respectively.^23−27^

An Innova (Bruker) atomic force microscope was used to determine the surface topography of methacrylic polymers based on 1-(4-nitrophenyl)piperazine thin films. The imaging tapping mode (with standard Si tips) was used during the measurements. The scan size was 2 μm × 2 μm. The roughness parameters (—average roughness and —root-mean-square roughness), characterizing the surface quality of the prepared thin layers were determined using NanoScope Analysis software (ver. 1.40).

Fourier transform infrared (FTIR) spectra were recorded in the spectral range from 400 to 1500 cm^–1^ using the spectrometer FT-IR Vertex 70 V. Raman spectra of thin films were also measured. We used a micro-Raman spectrometer (Senterra, Bruker Optik) with a λ = 785 nm laser, of about 20× optical zoom, and 50 mW laser power.

Electrochemical characterization was carried out by means of an Ivium Vertex potentiostat/galvanostat. CV was acquired using a three-electrode cell, where piperazine-based polymeric thin films were used as the working electrode and graphite wire and Ag/AgCl (3.0 M KCl) were the counter and reference electrodes, respectively. The scan rate was 100 mV/s starting from the open circuit potential. All the measurements were performed at room temperature in an aerated solution of 0.1 M tetrabutylammonium hexafluorophosphate (TBAPF6, for electrochemical analysis, 99%, Sigma Aldrich) in acetonitrile (CH_3_CN, HPLC Plus, ≥99.9% Sigma Aldrich).

### 2.3 Computational Details

**Figure 2:**
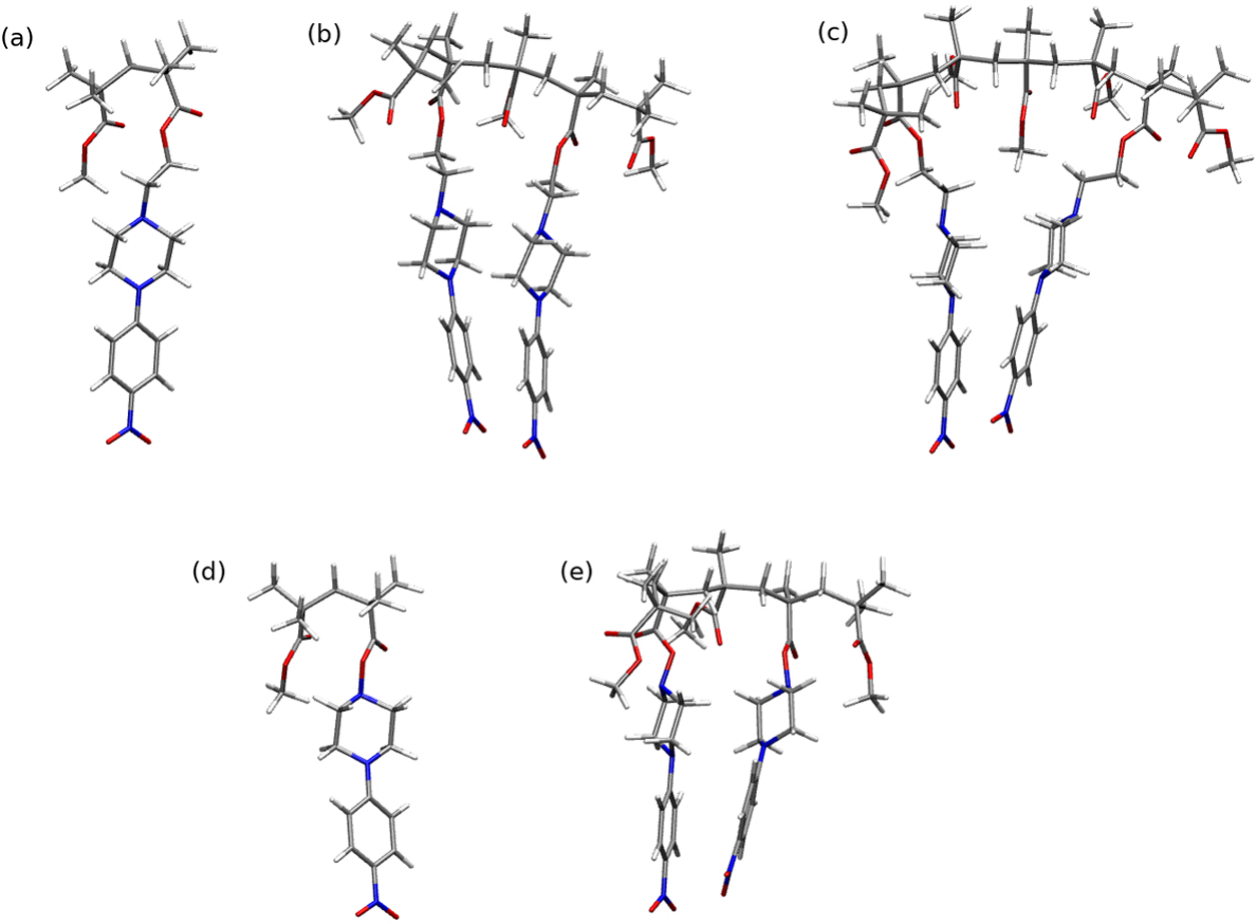
The ωB97X-D/def2-SVP(vacuum)-optimized geometries of the ground state of the models of analyzed systems: (a) OK1/OK3 unit, (b) OK1 dimer, (c) OK3 dimer, (d) OK2 unit, and (e) OK2 dimer.

The theoretical calculations have been performed for the model systems presented in Figure 2. The two molecules denoted further on as “monomers” (Figure 2a,d, for OK1/OK3 and OK2, respectively) were taken as the test systems for verification of the applied computational methodology and the initial investigation of the influence of the linker connecting the piperazine with the polymer chain. Furthermore, the so-called “dimers” are taken into account in order to analyze the effects imposed by the different distance of the chromophores in the polymer chain (see Figure 2b for OK1 dimer, (c) for OK3 dimer, and (e) for OK2 dimer). Full geometry optimization in vacuum has been performed for all of these structures with the ωB97X-D/def2-SVP approach. The character of the stationary points has been confirmed by harmonic vibrational analysis. The choice of a range-separated functional containing the posteriori dispersion correction has been rationalized by its overall good performance for the structural and spectroscopic characteristics of the organic dyes as well as for the vibronic coupling effects.^28^ The def2-SVP Weigend basis set, on the other hand, provides a reliable compromise between the quality of the obtained data and the computational costs. In order to verify the proper reproduction of the experimental absorption spectra, for the exemplary OK2 monomer model, other functionals known for their usefulness for the optical properties of the organic molecules have also been tested, namely, PBE0, M06-2X, and CAM-B3LYP. Moreover, the influence of the growing size of the basis set has been additionally confirmed by the application of the def2-TZVP basis set. All the approaches result only in tiny qualitative differences in the obtained data, which do not affect the discussion of the structural and absorption properties of the piperazine materials. For the sake of comparison, these data are provided in the Supporting Information, and the further discussion in the paper exploits only the ωB97X-D/def2-SVP approach.

Structural analysis of the dimers of the dyes attached to the polymer chain is supplemented by the non-covalent interaction analysis between the chromophore units performed with the NCIPlot program.^29−31^

The absorption spectrum has been estimated for monomers and dimers in a vertical approach in vacuum. The vibronic coupling arising from the S0 → S2 most intensive transition for the small illustrative example of OK2 monomer in the Franck–Condon and Franck–Condon–Herzberg–Teller regimes according to Barone et al.^32^ is also provided in order to verify the origin of the absorption band shapes. The harmonic IR and Raman spectra of the dimers of the analyzed systems, as well as the full set of frontier orbitals involved in the vertical transitions of the analyzed model systems, are given in the Supporting Information.

## 3 Results and Discussion

The copolymers OK1, OK2, and OK3 were obtained by radical polymerization of these monomers with MMA. Their structures were confirmed by ^1^H nuclear magnetic resonance (NMR) spectra and an accordance was observed between the observed n/m values in the polymers and the respective amounts of the introduced monomer ratios. The copolymerization ratio was calculated on the basis of the integration ratio of ^1^H NMR signals. Nevertheless, as anticipated for the resulting copolymers OK1, OK2, and OK3, the ratios of arylpiperazinyl to PMMA fragments were 1:1, 1:1, and 1:3, respectively. The structures of the synthesized copolymers are presented in Figure 1. The lower concentration of the arylpiperazinyl moiety, 25%, was obtained for copolymer OK3.

As expected, introduction of conformationally flexible alkyl chains within the nitrophenylpiperazine fragment tend to decrease *T*_g_ (OK1 vs OK2; OK3 vs OK2). The copolymer molecular weights (*M*_w_) are in the range of 32–35 kDa and polydispersity indices (*M*_w_/*M*_n_) are 1.6–1.85, as measured by GPC.

**Figure 3:**
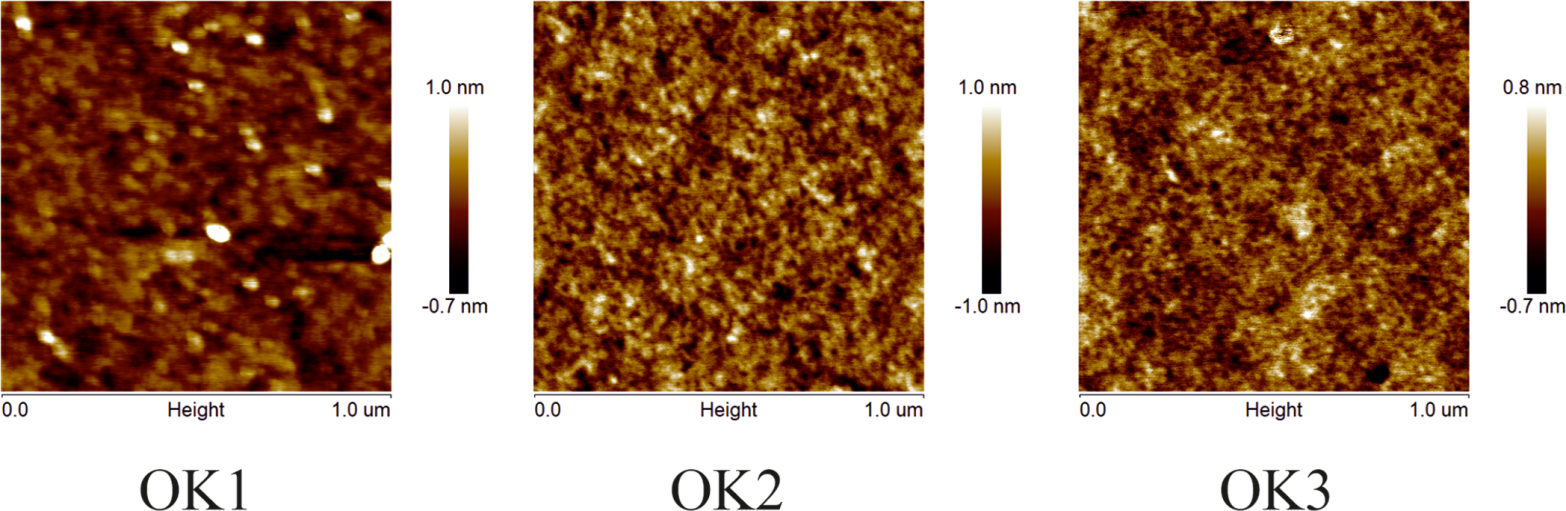
AFM images of OK1, OK2, and OK3 thin films.

Figure 3 shows the atomic force microscopy (AFM) images of three methacrylic polymers based on 1-(4-nitrophenyl)piperazine thin films. From these measurements, we determined the roughness parameters of the studied polymer thin films. We observed that the surfaces of all studied layers are very smooth. We found that the values of the roughness parameters (*R*_q_ and *R*_a_) are 0.239 and 0.162 nm for OK1, 0.285 and 0.226 nm for OK2, and 0.213 and 0.168 nm for OK3, respectively.

**Figure 4:**
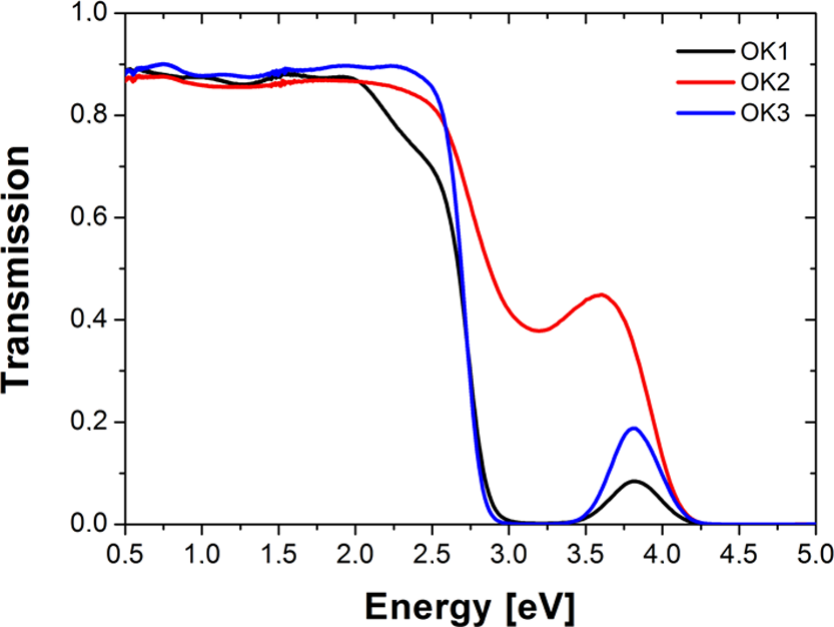
Transmission spectra of methacrylic polymers based on 1-(4-nitrophenyl)piperazine.

**Figure 5:**
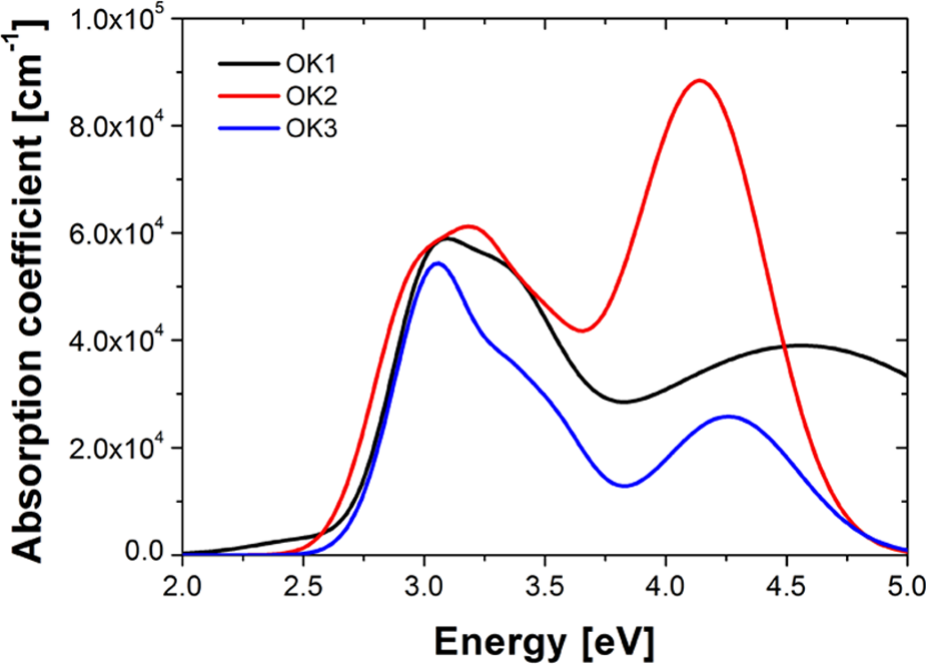
Absorption coefficient of methacrylic polymers based on 1-(4-nitrophenyl)piperazine.

Figure 4 presents the transmission spectra of the studied compounds. It can be seen that all studied compounds are non-absorbing materials up to about 2.0 eV, and above this value, they exhibit photon energy absorption, except the polymer with the longer chain (OK3). It should be mentioned that a similar behavior is also visible for the absorption coefficient of the studied polymers calculated from the spectroscopic ellipsometry (SE) measurements (see Figure 5). However, in Figure 4, there is only one visible absorption band in the transmission spectra of OK thin films due to the blocking of light by the glass. This band results from n–π* electronic transitions. In this region, it can be seen that after the addition of an ethylene group to the OK chain, there is a shift in the transmission spectrum toward higher energies and an increase in the bandwidth (see OK1 and OK3). In the region up to 2 eV for OK1 and OK3, we can see more oscillations than for OK2. This behavior is related to the thickness of the thin layer. Thus, the thickness of compounds with an additional ethylene group (OK1 and OK3) should be several times greater than for OK2. In the spectral range of 2.8–3.8 eV, the transmission of OK2 is about 0.4, which suggests that the OK2 thin film is thinner than OK1 and OK3. The thicknesses of the samples were confirmed by SE measurements (see Table 1).

**Table 1:** Values of Thickness (*L*), Optical Energy Band Gap (*E*_g_), and Energies of Inter-Band Transitions (*E*_1_–*E*_4_) Determined from SE Measurements

	OK1	OK2	OK3
*L* [nm]	772 ± 1	132 ± 1	1039 ± 1
*E*_g_ [eV]	2.80 ± 0.02	2.73 ± 0.02	2.81 ± 0.02
*E*_1_ [eV]	2.46 ± 0.03		
*E*_2_ [eV]	3.01 ± 0.01	2.98 ± 0.04	3.06 ± 0.01
*E*_3_ [eV]	3.40 ± 0.05	3.18 ± 0.07	3.47 ± 0.17
*E*_4_ [eV]	4.56 ± 0.07	4.14 ± 0.03	4.26 ± 0.03

From Figure 5, one can see that the absorption coefficient presents two absorption regions. The first band (from 2.0 to 3.7 eV) results from n–π* electronic transitions, whereas the UV region located in the 3.7–5.0 eV range is due to the vibronic coupling of π–π*. It should be mentioned that the shape of the absorption coefficient was parameterized using a sum of Gaussian oscillators. The interband transition energies of OK polymers are summarized in Table 1. These values refer to the center of the absorption bands.

From Figure 5, it can be seen that the addition of an ethylene group to the chain of OK causes the blue shift of the absorption coefficient spectra as well as a narrowing of the n–π* band and lowering of the π–π* band (see OK1 and OK3). Besides, the n–π* band for all compounds does not change much. However, there are certain characteristics that distinguish between these materials in this region. The peaks at 3.06 eV for OK3 and 3.01 eV for OK1, that is, compounds with an additional ethylene group, are more visible than in the case of OK2 (compound without the ethylene group and as a result with a shorter side-chain in the polymer). On the other hand, the peak at about 3.18 eV is clearly visible for OK2. In the case of OK1 and OK3, there are shoulders at about 3.40 eV and 3.47 eV, respectively, which have been blue-shifted compared to OK2 (3.18 eV) due to the additional ethylene group. Moreover, the absorption coefficient spectra of the compound without the ethylene group (OK2) is narrow and has the highest value in the π–π* region. The sample with the ethylene group and a longer polymer chain (OK3) possesses a similar shape to the compound without the ethylene group (OK2) and a similar absorption coefficient in this area. However, the addition of the ethylene group and elongation of the polymer chain cause a reduction in its absorption coefficient. In the case of OK1, that is, the compound with the ethylene group and a 1:1 n/m ratio, this band is broadened and flattened.

From Figure 5, one can see that the addition of the ethylene group and elongation of the polymer chain of compound (see OK3) reduce the value of the absorption coefficient. Whereas the additional ethylene group in OK compound decreases the value of the absorption coefficient only in the π–π* region.

**Figure 6:**
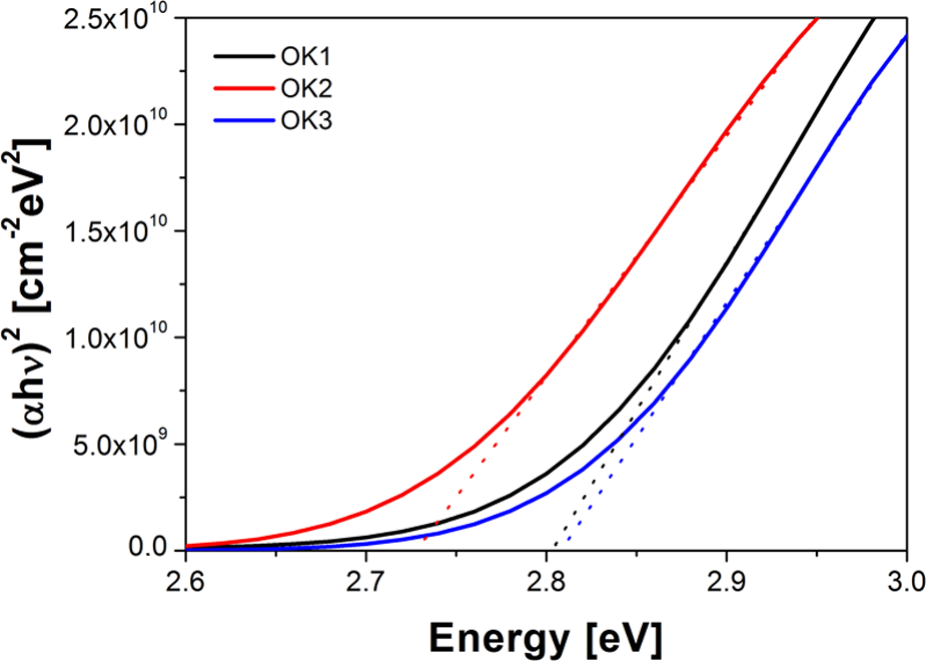
Tauc’s plot of methacrylic polymers with a 1-(4-nitrophenyl)piperazine fragment.

To determine the optical energy band gap (*E*_g_), we plotted (α*h*ν)^2^ as a function of energy and then we fitted the linear dependence according to Tauc’s procedure (see Figure 6). The presented fits show the existence of direct allowed band gaps for all studied polymers. The *E*_g_ values of the studied polymers are presented in Table 1, and they change as follows: OK2 < OK1 < OK3. Thus, the compound without the ethylene group and with a shorter side-chain in the polymer has the lowest value of *E*_g_. On the other hand, the sample with an alkyl chain has the highest value of optical energy band gap.

**Figure 7:**
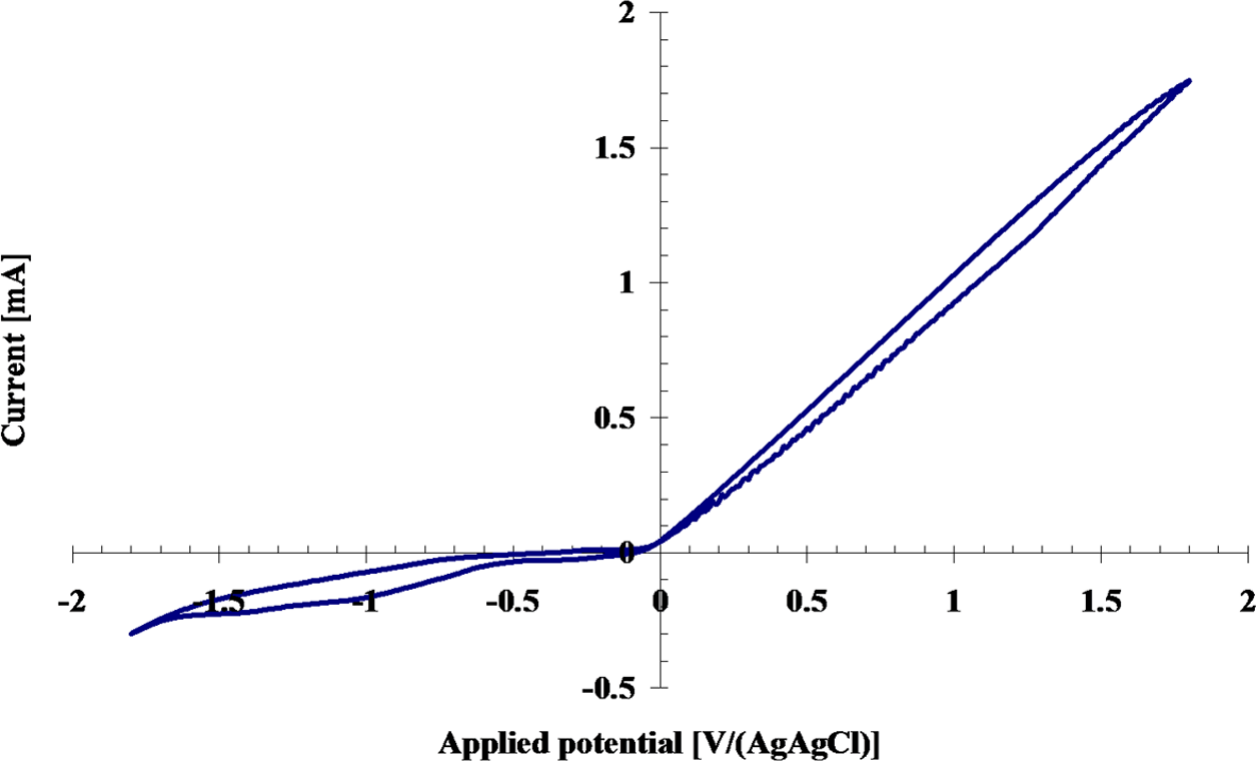
CV curve of the OK2 compound.

Electrochemical investigations evidenced a negligible redox response of 1-(4-nitrophenyl)piperazine containing methylene groups, despite the length of the polymer. These polymers showed just an irreversible cathodic peak at around −1.0 V/AgAgCl. An evident redox activity was shown by the OK2 compound, as displayed in Figure 7.

OK2 showed an oxidation potential onset at −0.03 V/AgAgCl and a reduction potential onset at −0.441 V/AgAgCl. In addition, an irreversible cathodic peak was evidenced at around −1.0 V/AgAgl, as in the cases of OK1 and OK3. The irreversible cathodic peak, common to all the OK compounds, can be assigned to 4-nitrophenyl reduction, in agreement with literature.^33^

The oxidation potential onset is related to the IP according to IP = *E*_onset_^ox^ + 4.5, where *E*_onset_^ox^ is the oxidation potential onset with respect to the normal hydrogen electrode.^21^ Consequently, an IP value of 4.67 eV was calculated for OK2. According to Koopmans’ theorem, the highest occupied molecular orbital (HOMO) energy is equal to the negative IP energy value.^34^ Thus, the HOMO level of OK2 was located at −4.67 eV with respect to the vacuum level. By considering that the optical energy gap represents the distance between the HOMO and LUMO levels, its LUMO level was located at −1.94 eV.

**Figure 8:**
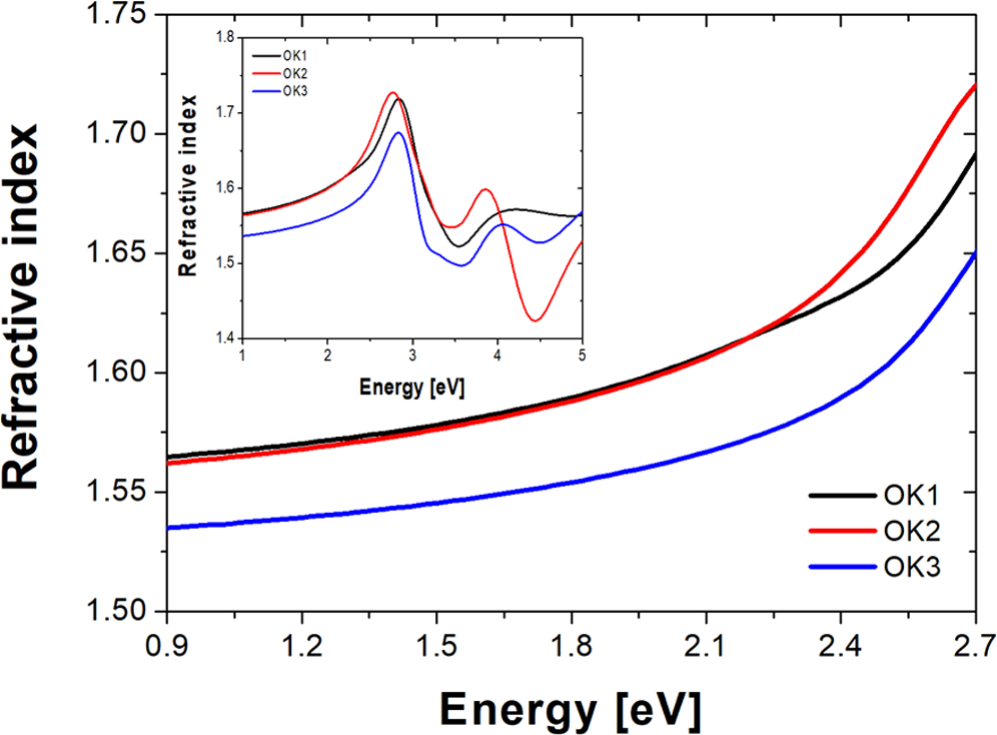
Refractive index of methacrylic polymers based on 1-(4-nitrophenyl)piperazine.

It is well known that the knowledge of the refractive index (n) is very important in the design of optical devices. The dependence of the refractive index as a function of energy is shown in Figure 8. It can be seen that the refractive indices of studied compounds increase continuously as the energy increases up to 2.7 eV. It means that n shows a normal dispersion in this region, and this behavior can be described using a well-known Sellmeier relation. Above 2.7 eV, we have the anomalous dispersion (see the inset of Figure 8). The values of the refractive index are almost similar for compounds with the ethylene group (OK1) and without (OK2) in the region of 1–2.3 eV. Whereas, the n values of OK2 are slightly higher compared with OK1 from 2.3 to 2.7 eV. In the case of compound with the ethylene group and a longer polymer chain (OK3), the values of its refractive index are lower than those in other studied samples. This behavior may be due to the presence of a longer polymer chain as well as the refractive index decrease with the decrease of the concentration of the arylpiperazinyl moiety in copolymers (OK1 and OK2 vs OK3; n/m ratio 1:1 vs n/m ratio 1:3).

**Figure 9:**
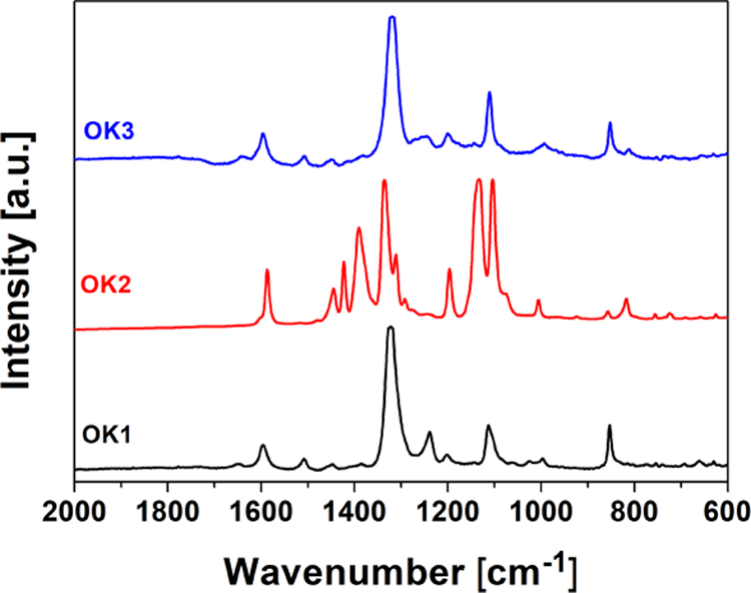
Raman spectra of OK1–OK3 thin films.

Figure 9 shows the Raman plots for OK polymer thin films. It should be noted that the spectra for OK1 and OK3 samples are very similar. Whereas the spectrum of OK2 polymer differs significantly from the other two due to the difference in the chemical structure of the compound, that is, the absence of the −CH_2_–CH_2_– group linking the polymer chain and the piperazine ring. The bands at 853, 1100, and 1236 cm^–1^ observed in all registered spectra are typical for the PMMA spectrum and can be assigned to δ(CH_2_), ν(C–C) skeletal mode, and the first overtone involving (C=O) of C–COO, respectively. The characteristic band at around 1320 cm^–1^ was derived from the NO_2_ symmetric stretch and the band at around 1585 cm^–1^ was from the NO_2_ asymmetric stretch.^35^ Additionally, for the sample OK2, a strong band at 1390 cm^–1^ is observed. Moreover, the in the case of NO_2_, bands occur as doublets (1107 and 1134 cm^–1^; 1310 and 1423 cm^–1^).

**Figure 10:**
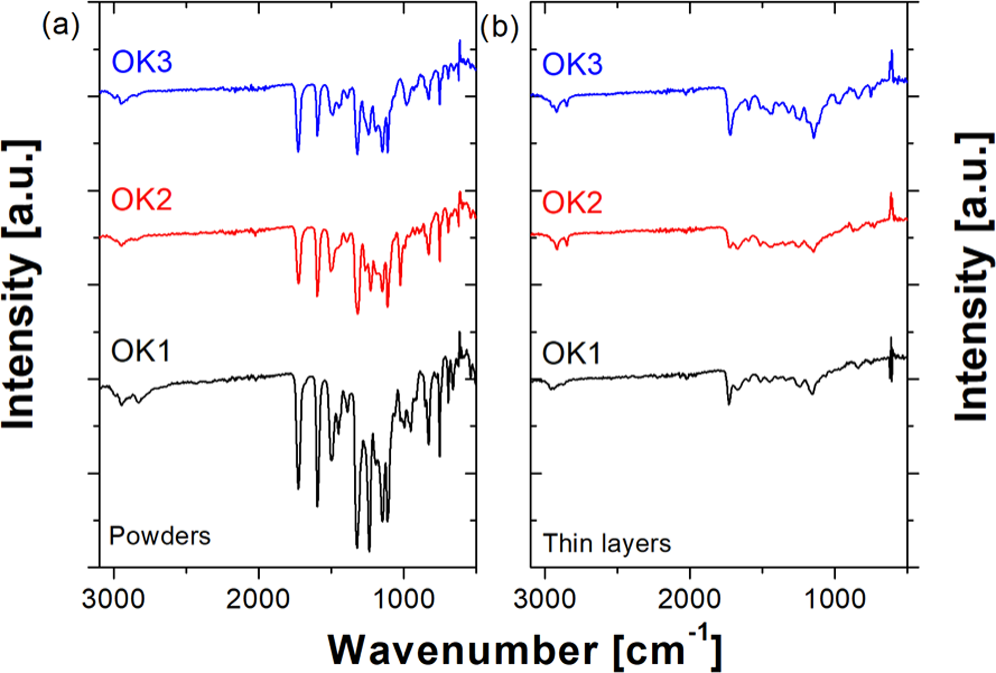
FTIR spectra of methacrylic polymers based on 1-(4-nitrophenyl)piperazine measured as powders (a) and thin layers (b).

Figure 10 shows the FTIR spectra of powders and fabricated films of OK1, OK2, and OK3 samples. Comparing the IR spectra presented in Figure 10a,b, we can see that the polymerization process has taken place. The main evidence to prove that the monomers have been copolymerized is the disappearance of the absorption peaks at 940 cm^–1^, which are assigned to the C–H bond derived from the C=CH_2_ group and the stretching vibration band of C=C at 1670 cm^–1^. Four characteristic bands at 1720, 1520, 1330, and 1158 cm^–1^ are visible in all the spectra of the deposited layers, which can be assigned to the carbonyl group (C=O), nitro group (ν_as_(NO_2_) and ν_s_(NO_2_) vibrations), and ether group (C–O–C), respectively.

**Figure 11:**
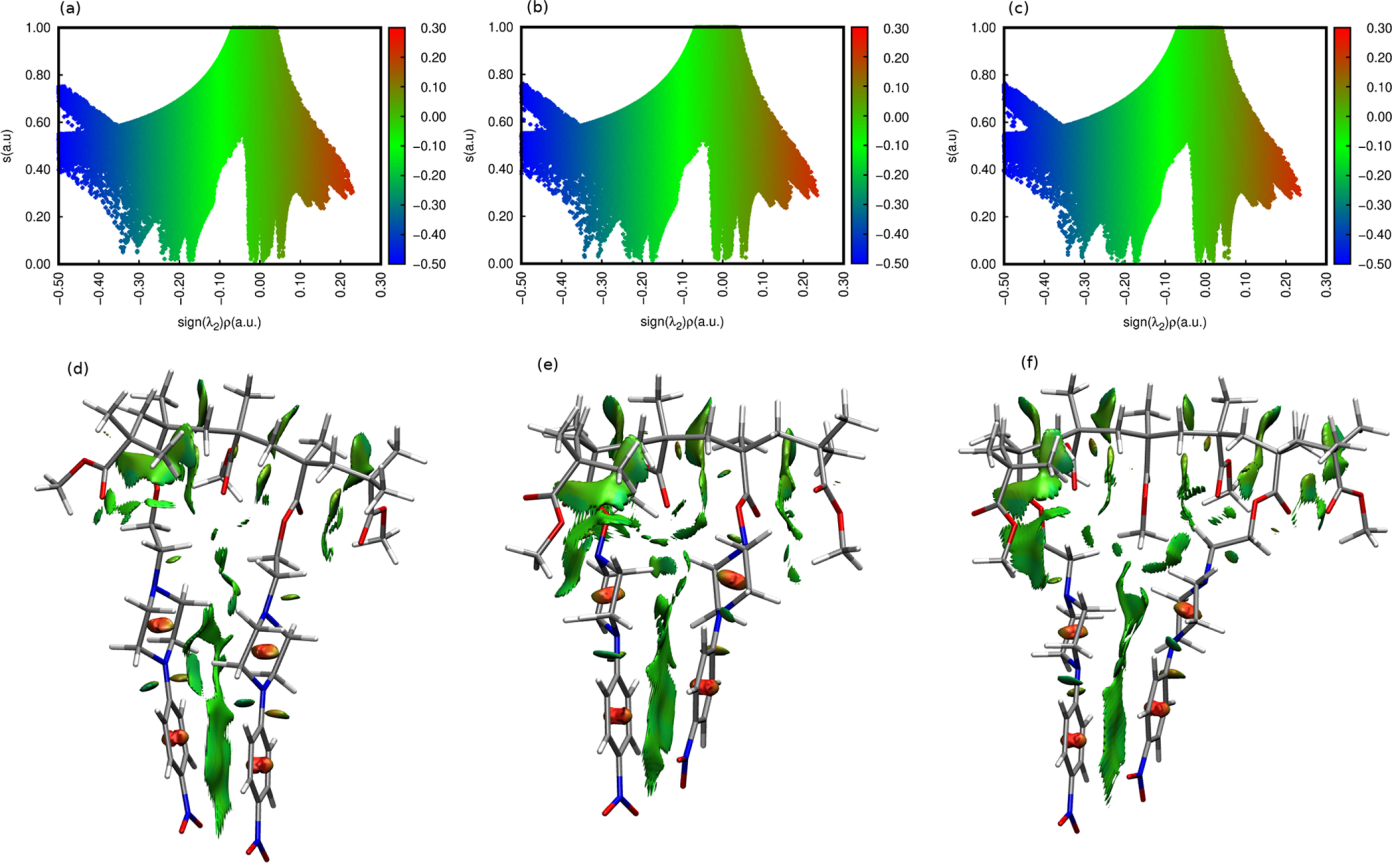
Non-covalent interactions for OK1, OK2, and OK3 dimers presented in Figure 2. (ωB97X-D/def2-SVP) according to NCIPlot analysis. Panels (a)–(c) present the relationship between reduced density gradient (s) and the sign of the second eigenvalue (λ2) of the promolecular density. The spikes close to 0 correspond to the van der Waals interactions and correspond to the NCI surfaces, depicted in (d)–(f) with gradient green color.

The optimized dimer structures for OK1, OK2, and OK3, presented in Figure 2, clearly show that the chromophoric units incorporated into the polymer chain tend to arrange themselves in a parallel way in order to maximize the mutual dispersion π–π interactions. Neither the length of the linker nor the distance between the dye moieties in the chain affect significantly the alignment of the piperazine units. In all cases, they tend to approach one another, exploiting the flexibility of the polymer chain and the linker. Therefore, the intramolecular interactions inside the polymeric chain are similar in all of the analyzed systems, which is clearly confirmed by the non-covalent interactions presented in Figure 11.

**Figure 12:**
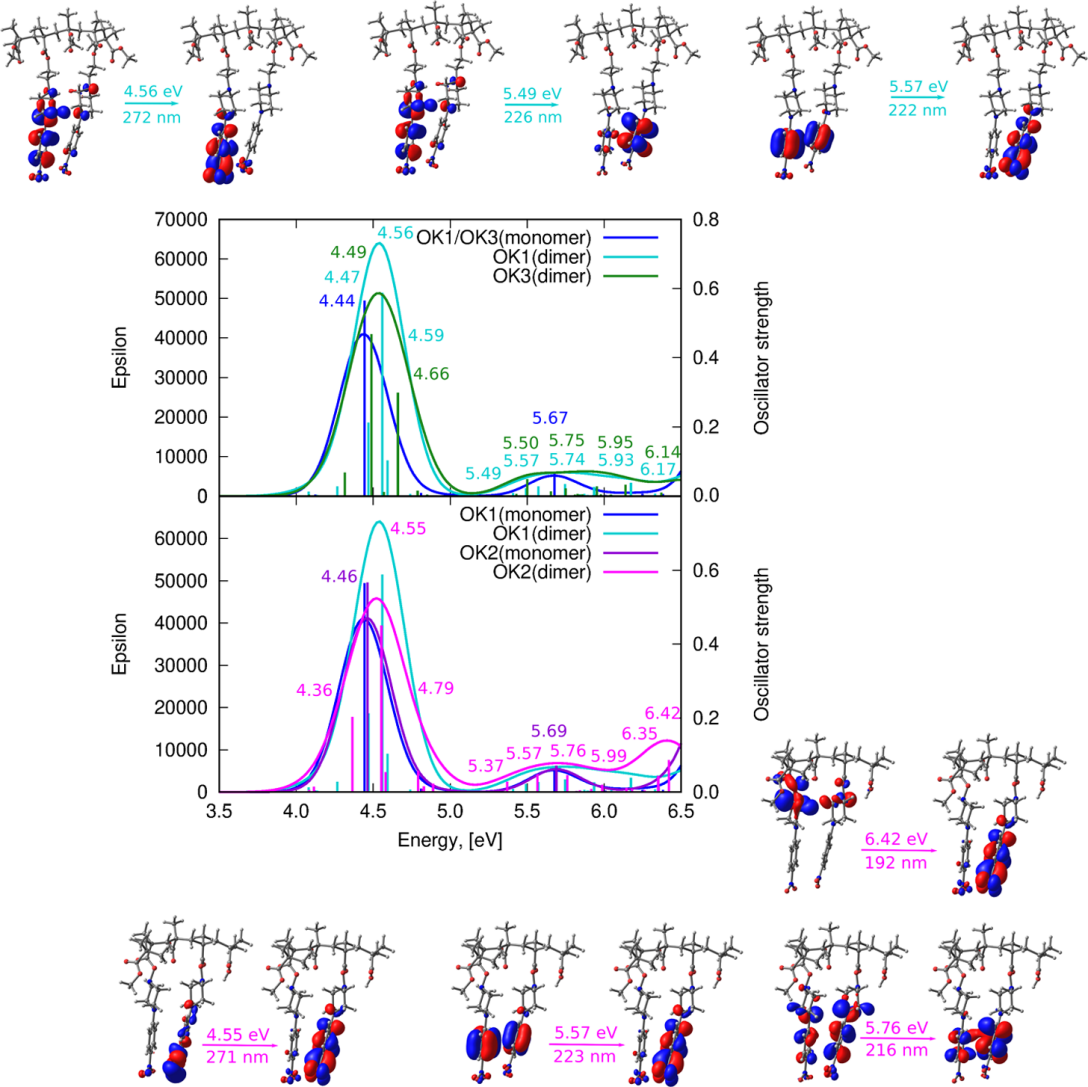
Theoretical (ωB97X-D/def2-SVP)absorption spectra for OK1, OK2, and OK3 models presented in Figure 2 together with the frontier molecular orbitals of OK1 and OK2 dimers involved in most intensive transitions in both ranges. Upper panel presents the influence of the distance between the chromophore units in the polymer chain and the lower panel compares the influence of the length of the linker between the chromophore and the polymer chain.

The vertical absorption spectrum calculated within the ωB97X-D/def2-SVP approach is presented in Figure 12, together with the most important frontier orbitals for OK1 and OK2 dimers. Due to the shortcomings of the applied models (such as model simplifications, intrinsic DFT limitations, or the vertical approximation applied), a significant shift of the absorption bands in the direction of higher energies with respect to the experiment is noticed (for instance, theoretical 4.56 eV for OK1 dimer vs about 3.0 eV in the experiment). However, the qualitative shape of the bands and the relative positions of the two most intensive transitions in the computational absorption spectrum reproduces well the tendencies observed in the experiment. The estimation of the quality of the applied approaches has been performed and is summarized in Supporting Information (Figures S1 and S2). The position and the shape of the spectrum for the exemplary system, namely, OK2 monomer, calculated with different functionals and basis sets are presented in Figure S1. The qualitative agreement of the spectral shape with experimental findings is achieved in all of the applied approaches, while the shift of the signal arising from the increase of the basis set from double- to triple-zeta is not larger than 0.25 eV. However, in Figure S1, one can clearly notice that the selected functional can affect the position of the most intensive band, and the corresponding shift can be as large as 0.5 eV (4.49 eV at M06-2X/def2-SVP vs 4.01 eV for the PBE0/def2-SVP approach). On the other hand, the spectral shift arising from the application of the Franck–Condon principle within the vertical approximation at the ωB97X-D/def2-SVP level is presented for the model OK2 monomer in Figure S2. The vertical S0 → S2 transition is estimated to occur at 4.46 eV, while the corresponding value for the adiabatic transition (between two minima on the potential energy surfaces for S0 and S2 states, respectively) appears at 4.24 eV, and the introduction of the zero-point vibrational energy correction further improves the result, giving the energy value equal to 4.10 eV. Thus, the provided theoretical results should be treated as the qualitative explanation of the nature of the experimentally observed transitions.

All the investigated systems exhibit the two bands: one of higher intensity, below 5 eV, and the other, less intensive, between 5 and 6 eV. The orbitals involved in these transitions (the selected ones are presented in Figure 12 and the remaining part is depicted in the Supporting Information) show the moderate charge transfer from the nitrogen atom in the piperazine moiety to the terminal −NO_2_ group, confirming their n → π* character. In contrast, the high-energy bands originate mostly from the transitions of the π → π* type localized either on a single monomer or delocalized between the chromophoric units of adjacent monomers.

## Conclusions

The three novel methacrylic side-chain polymers based on 1-(4-nitrophenyl)piperazine are obtained and characterized with respect of their photo-physical properties for optoelectronic applications. The surface, structural, and optical properties of the analyzed materials change with the increase of the chromophore distance from the polymer main chain by adding an ethylene group and also with the growth of the separation between the chromophoric units in the polymer. The experimentally obtained results were confirmed by the theoretical DFT calculations, which reveal the similarity of all of the investigated systems, but yet the possibility of precise tuning of their photo-optical properties by the proposed structural modifications.

We found that adding the ethylene group to the OK chain causes the blue shift of the absorption spectrum, as well as narrowing of the n–π* band and lowering of the intensity of the π–π* band. It was shown that the lowest refractive index values are for the compound with the ethylene group and the longer polymer chain. Refractive index decreases with a decrease of concentration of 1-(4-nitrophenyl)piperazine fragment in the copolymer. It also turned out that the investigated piperazine-containing polymers have a low optical energy band gap, which increases slightly after the addition of the ethylene group to their chain. Such behavior makes them suitable materials for their use in optoelectronic devices due to the possibility of modulating their energy band gap by modifying their structure. Since the greater efficiency in commercial devices can be obtained when the optical energy band gap is in the visible region of the spectrum, all the studied piperazine-containing polymers exhibit semiconductor behavior (*E*_g_ in the range from 2.73 to 2.81 eV), thus they can be used in optoelectronic applications (e.g., photovoltaic devices). The present basic research considering the influence of the structural and electronic factors on the optical properties of new piperazine-based materials seems to provide promising results in the field of optoelectronic materials and requires further thorough investigations including the parameters of crucial importance for real applications, such as electron affinity, IPs, current density, open circuit voltage, shunt resistance, fill factor, and power efficiency. Thus, the present study delivers the valuable alternative for the widely applied modifications of the PMMA chains with the popular chromophores such as azobenzenes, providing the enhancement of the properties essential for the construction of practical devices.

## Supporting Information Available

Verification of the approach applied for the calculations of the absorption spectrum, the vibronic effects for the OK2 monomer, frontier molecular orbitals for OK1 and OK2 monomers and all dimers, theoretical IR and Raman spectra, and numerical values of absorption energy, wavelength, and oscillator strength (PDF)

## References

[ref1] Shepherd W. E. B.; Platt A. D.; Hofer D.; Ostroverkhova O.; Loth M.; Anthony J. E. Aggregate formation and its effect on (opto)electronic properties of guest-host organic semiconductors. Appl. Phys. Lett. 2010, 97, 163303 10.1063/1.3505493.

[ref2] Zhang Z.; Edkins R. M.; Nitsch J.; Fucke K.; Eichhorn A.; Steffen A.; Wang Y.; Marder T. B. D-π-A Triarylboron Compounds with Tunable Push-Pull Character Achieved by Modification of Both the Donor and Acceptor Moieties. Chem.—Eur. J. 2015, 21, 177–190. 10.1002/chem.201405621. 25413782

[ref3] Gong Y.; Tan Y.; Liu J.; Lu P.; Feng C.; Yuan W. Z.; Lu Y.; Sun J. Z.; He G.; Zhang Y. Twisted D−π–A solid emitters: efficient emission and high contrast mechanochromism. Chem. Commun. 2013, 49, 4009–4011. 10.1039/c3cc39243k. 23563355

[ref4] Achelle S.; Plé N.; Turck A. Incorporation of pyridazine rings in the structure of functionalized π-conjugated materials. RSC Adv. 2011, 1, 364–388. 10.1039/c1ra00207d.

[ref5] Tirelli N.; Altomare A.; Solaro R.; Ciardelli F.; Follonier S.; Bosshard C.; Günter P. Structure–activity relationship of new NLO organic materials based on push–pull azodyes. 4. Side chain polymers. Polymer 2000, 41, 415–421. 10.1016/s0032-3861(99)00202-5.

[ref6] Marin L.; Zabulica A.; Moleavin I.-A. Luminescent guest–host composite films based on an azomethine dye in different matrix polymers. Opt. Mater. 2014, 38, 290–296. 10.1016/j.optmat.2014.10.056.

[ref7] Figà V.; Chiappara C.; Ferrante F.; Casaletto M. P.; Principato F.; Cataldo S.; Chen Z.; Husta H.; Usta A.; Pignataro B. Symmetric Naphthalenediimidequaterthiophenes for Electropolymerized Electrochromic Thin films. J. Mater. Chem. C 2015, 3, 5985–5994. 10.1039/c5tc00746a.

[ref8] Prabavathi N.; Senthil Nayaki N.; Krishnakumar V. Spectroscopic Investigation (FT-IR, FT-Raman, NMR and UV-Vis), Conformational Stability, NBO and Thermodynamic Analysis of 1-(2-Methoxyphenyl) Piperazine and 1-(2-Chlorophenyl) Piperazine by DFT Approach. Pharm. Anal. Acta 2015, 06, 1000391 10.4172/2153-2435.1000391.

[ref9] Prabavathi N.; Nilufer A.; Krishnakumar V. FT-IR, FT-Raman and DFT quantum chemical study on the molecular conformation, vibrational and electronic transitions of 1-(m-(trifluoromethyl)phenyl)piperazine. Spectrochim. Acta, Part A 2014, 121, 483–493. 10.1016/j.saa.2013.10.102. 24291424

[ref10] Zhang M.; Zeng G.; Liao X.; Wang Y. An antibacterial and biocompatible piperazine polymer. RSC Adv. 2019, 9, 10135–10147. 10.1039/c9ra02219h. PMC9062374 35520902

[ref11] Stewart D. J.; Long S. L.; Yu Z.; Kannan R.; Mikhailov A.; Rebane A.; Tan L. S.; Haley J. E. The fluorescence of a chelating two-photon-absorbing dye is enhanced with the addition of transition metal ions but quenched in the presence of acid. Proc. SPIE 2016, 9939, 993904 10.1117/12.2236658.

[ref12] Bhat M. A.; Lone S. H.; Butcher R. J.; Srivastava S. K. Theoretical and experimental investigations into structural, electronic, molecular and biological properties of 4-(3-chlorophenyl)-1-(3-chloropropyl) piperazin-1-ium chloride. J. Mol. Struct. 2018, 1168, 242–249. 10.1016/j.molstruc.2018.05.019.

[ref13] Hurenkamp J. H.; Browne W. R.; Augulis R.; Pugžlys A.; van Loosdrecht P. H. M.; van Esch J. H.; Feringa B. L. Intramolecular energy transfer in a tetra-coumarin perylene system: influence of solvent and bridging unit on electronic properties. Org. Biomol. Chem. 2007, 5, 3354–3362. 10.1039/b711681k. 17912390

[ref14] Yan L.; Wang Y.; Wei J.; Ji G.; Gu H.; Li Z.; Zhang J.; Luo Q.; Wang Z.; Liu X.; et al. Simultaneous performance and stability improvement of polymer:fullerene solar cells by doping with piperazine. J. Mater. Chem. A 2019, 7, 7099–7108. 10.1039/c8ta12109e.

[ref15] Yan L.; Gu H.; Li Z.; Zhang J.; Yang Y.; Wang H.; Liu X.; Wei Z.; Luo Q.; Ma C.-Q. The interfacial degradation mechanism of polymer:fullerene bis-adduct solar cells and their stability improvement. Mater. Adv. 2020, 1, 1307–1317. 10.1039/d0ma00277a.

[ref16] Zhang C.; Heumueller T.; Leon S.; Gruber W.; Burlafinger K.; Tang X.; Perea J. D.; Wabra I.; Hirsch A.; Unruh T.; et al. A Top-down Strategy Identifying Molecular Phase Stabilizers to Overcome Microstructure Instabilities in Organic Solar Cells. Energy Environ. Sci. 2019, 12, 1078–1087. 10.1039/c8ee03780a.

[ref17] Do J.; Huh J.; Kim E. Solvatochromic Fluorescence of Piperazine-Modified Bipyridazines for an Organic Solvent-Sensitive Film. Langmuir 2009, 25, 9405–9412. 10.1021/la901476q. 19601612

[ref18] Kubo K.; Hayakawa A.; Sakurai T.; Igarashi T.; Matsumoto T.; Takahashi H.; Takechi H. Crystal Structure and Complexation and Fluorescence Behaviors of 1,4-Bis (9-anthracenylmethyl) piperazine. J. Oleo Sci. 2010, 59, 661–666. 10.5650/jos.59.661. 21099144

[ref19] Prabavathi N.; Nilufer A.; Krishnakumar V. FT-Raman and DFT quantum chemical study on the molecular conformation, vibrational and electronic transitions of 1-(m-(trifluoromethyl)phenyl)piperazine. Spectrochim. Acta, Part A 2014, 121, 483–493. 10.1016/j.saa.2013.10.102. 24291424

[ref20] Wcisło A.; Dąbkowska I.; Czupryniak J.; Ossowski T.; Zarzeczańska D. Unusual behavior in di-substituted piperidine and piperazine anthraquinones upon protonation – Spectral, electrochemical, and quantum chemical studies. J. Mol. Liq. 2019, 279, 154–163. 10.1016/j.molliq.2019.01.115.

[ref21] Cervini R.; Li X.-C.; Spencer G. W. C.; Holmes A. B.; Moratti S. C.; Friend R. H. Electrochemical and optical studies of PPV derivatives and poly(aromatic oxadiazoles). Synth. Met. 1997, 84, 359–360. 10.1016/s0379-6779(97)80781-3.

[ref22] Wienk M. M.; Turbiez M. G. R.; Struijk M. P.; Fonrodona M.; Janssen R. A. J. Low-band gap poly(di-2-thienylthienopyrazine):fullerene solar cells. Appl. Phys. Lett. 2006, 88, 153511 10.1063/1.2195897.

[ref23] Derkowska-Zielinska B.; Skowronski L.; Biitseva A.; Grabowski A.; Naparty M. K.; Smokal V.; Kysil A.; Krupka O. Optical characterization of heterocyclic azo dyes containing polymers thin films. Appl. Surf. Sci. 2017, 421, 361–366. 10.1016/j.apsusc.2016.12.080.

[ref24] Derkowska-Zielinska B.; Skowronski L.; Kozlowski T.; Smokal V.; Kysil A.; Biitseva A.; Krupka O. Influence of peripheral substituents on the optical properties of heterocyclic azo dyes. Opt. Mater. 2015, 49, 325–329. 10.1016/j.optmat.2015.10.001.

[ref25] Skowronski L.; Krupka O.; Smokal V.; Grabowski A.; Naparty M.; Derkowska-Zielinska B. Optical properties of coumarins containing copolymers. Opt. Mater. 2015, 47, 18–23. 10.1016/j.optmat.2015.06.047.

[ref26] Derkowska-Zielinska B.; Krupka O.; Smokal V.; Grabowski A.; Naparty M.; Skowronski L. Optical properties of disperse dyes doped poly(methyl methacrylate). Mol. Cryst. Liq. Cryst. 2016, 639, 87–93. 10.1080/15421406.2016.1254585.

[ref27] Chomicki D.; Kharchenko O.; Skowronski L.; Kowalonek J.; Kozanecka-Szmigiel A.; Szmigiel D.; Smokal V.; Krupka O.; Derkowska-Zielinska B. Physico-chemical and light-induced properties of quinoline azo-dyes polymers. Int. J. Mol. Sci. 2020, 21, 5755 10.3390/ijms21165755. PMC7461123 32796673

[ref28] Jacquemin D.; Brémond E.; Planchat A.; Ciofini I.; Adamo C. TD-DFT Vibronic Couplings in Anthraquinones: From Basis Set and Functional Benchmarks to Applications for Industrial Dyes. J. Chem. Theory Comput. 2011, 7, 1882–1892. 10.1021/ct200259k. 26596449

[ref29] Boto R. A.; Peccati F.; Laplaza R.; Quan C.; Carbone A.; Piquemal J.-P.; Maday Y.; Contreras-Garcia J. NCIPLOT4: A new step towards a fast quantification of noncovalent interactions. https://github.com/juliacontrerasgarcia/nciplot (Last accessed 19 4, 2021).

[ref30] Johnson E. R.; Keinan S.; Mori-Sánchez P.; Contreras-García J.; Cohen A. J.; Yang W. Revealing Noncovalent Interactions. J. Am. Chem. Soc. 2010, 132, 6498–6506. 10.1021/ja100936w. 20394428 PMC2864795

[ref31] Contreras-García J.; Johnson E. R.; Keinan S.; Chaudret R.; Piquemal J.-P.; Beratan D. N.; Yang W. NCIPLOT: A Program for Plotting Noncovalent Interaction Regions. J. Chem. Theory Comput. 2011, 7, 625–632. 10.1021/ct100641a. 21516178 PMC3080048

[ref32] Barone V.; Bloino J.; Biczysko M. Vibrationally Resolved Electronic Spectra in Gaussian09; Gaussian, White Paper 2009.

[ref33] Sunday C.; Masikini M.; Wilson L.; Rassie C.; Waryo T.; Baker P.; Iwuoha E. Application on Gold Nanoparticles-Dotted 4-Nitrophenylazo Graphene in a Label-Free Impedimetric Deoxynivalenol Immunosensor. Sensors 2015, 15, 3854–3871. 10.3390/s150203854. 25668213 PMC4367389

[ref34] Koopmans T. Über die Zuordnung von Wellenfunktionen und Eigenwerten zu den einzelnen Elektronen eines Atoms. Physica 1934, 1, 104–113. 10.1016/s0031-8914(34)90011-2.

[ref35] Shakila G.; Saleem H.; Sundaraganesan N. FT-IR, FT-Raman, NMR and U-V Spectral investigation: Computation of vibrational frequency, chemical shifts and electronic structure calculations of 1-bromo-4-nitrobenzene. World Sci. News 2017, 61, 150–185.

