# Supplementary material for: Distance Effects of Phenylpiperazine-Containing Methacrylic Polymers on Optical and Structural Properties
Source: J Phys Chem B. 2021 Sep 9;125(37):10629–38. doi: 10.1021/acs.jpcb.1c05654 (PMC8474111; doi:10.1021/acs.jpcb.1c05654)
Supplement: Supplementary file 1 — jp1c05654_si_001.pdf [file jp1c05654_si_001.pdf]

Distance Effects of Phenylpiperazine Containing Methacrylic Polymers on Optical and Structural Properties

Beata Derkowska-Zielinska[1,], Anna Kaczmarek-Kedziera[2], Malgorzata Sypniewska[1], Dariusz Chomicki[1], Robert Szczesny[2], Lukasz Skowronski[3], Viviana Figà[4], Oksana Krupka[5]*

[1]Institute of Physics, Faculty of Physics, Astronomy and Informatics, Nicolaus Copernicus University in Torun, Grudziadzka 5, 87-100 Torun, Poland

[2]Faculty of Chemistry, Nicolaus Copernicus University in Torun, Gagarina 7, 87-100 Torun, Poland

[3]Institute of Mathematics and Physics, UTP University of Science and Technology, S. Kaliskiego 7, 85-796 Bydgoszcz, Poland

[*] Corresponding author: Beata Derkowska-Zielinska, e-mail address: beata@fizyka.umk.pl
Institute of Physics, Faculty of Physics, Astronomy and Informatics, Nicholas Copernicus University in Torun, Grudziadzka 5/7, 87-100 Torun, Poland

[4]Euro-Mediterranean Institute of Science and Technology Palermo, via Michele Miraglia 20, 90100, Palermo, Italy

[5]Taras Shevchenko National University of Kyiv, 64/13 Volodymyrska St., 01601 Kyiv, Ukraine

Supplementary Information:

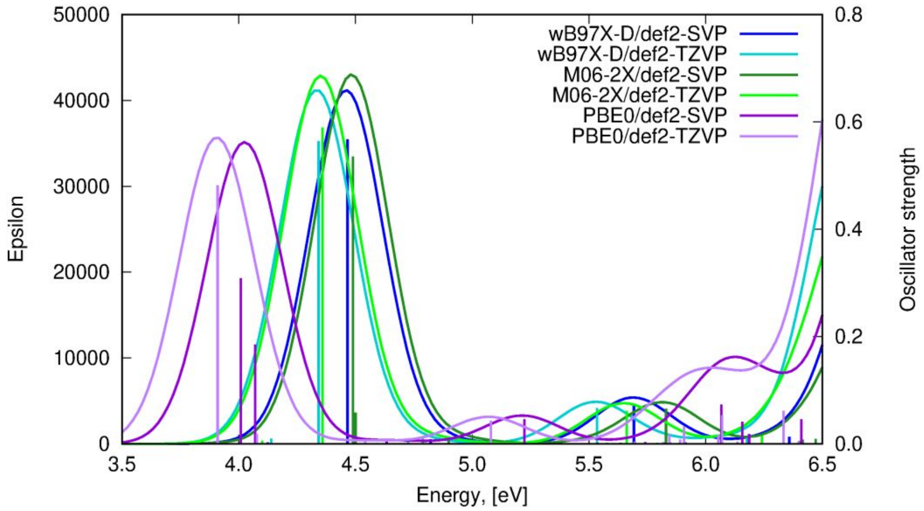

Figure S1. Performance of the applied approaches for the vertical absorption spectrum of OK2 unit (blue curves for ωB97X-D functional, green for M06-2X functional, violet for PBE0 functional, dark lines for def2-SVP basis set and light lines of each color – for def2-TZVP basis set). The shifts of the absorption bands with the modification of the approach can be as large as 0.5 eV for different functionals or of the order of 0.2 eV for different basis sets, however the qualitative shape

and relative bands positions are well reproduced, independently on the functional and basis set applied.

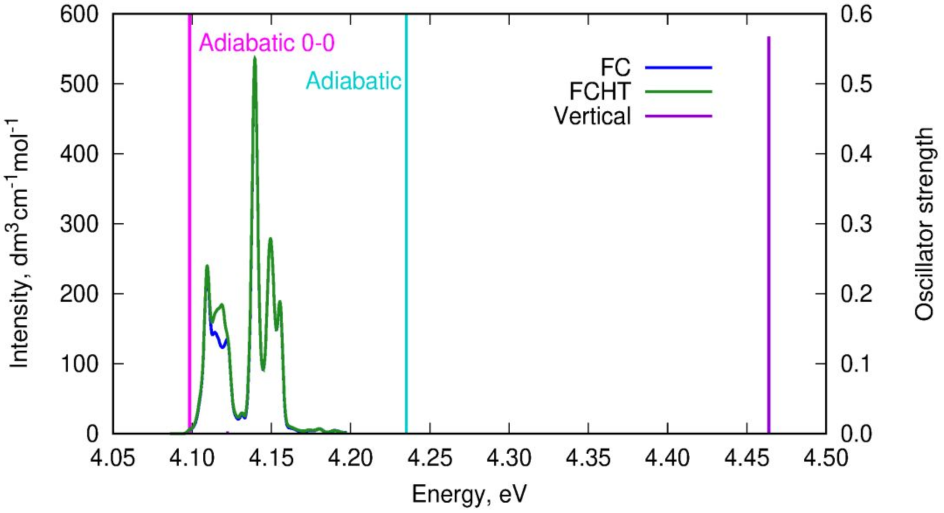

Figure S2. Comparison of the theoretical vertical signal (dark violet), adiabatic transition (turquoise) and adiabatic 0-0 transition (magenta) with the Franck-Condon (blue, FC) and Franck-Condon-Herzberg-Teller (green, FCHT) vibronic effects for the S0→S2 transition of OK2 unit within the ωB97X-D/def2-SVP approach

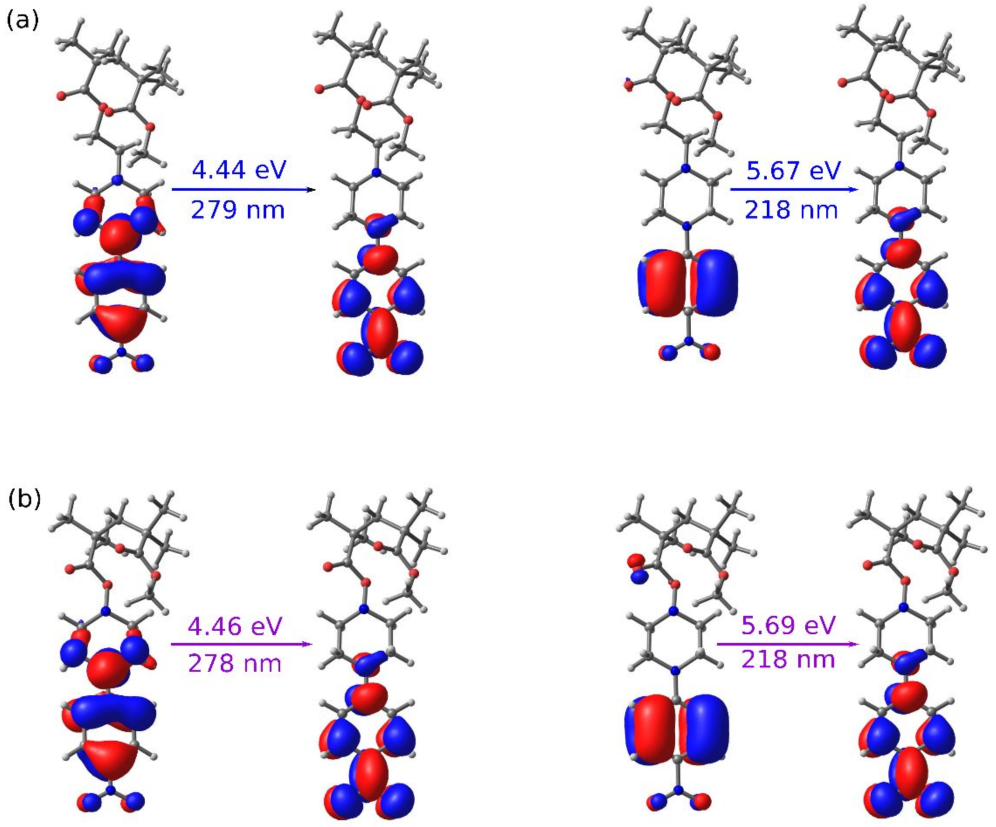

Figure S3. Frontier molecular orbitals of OK1 (panel (a)) and OK2 (panel (b)) units (the corresponding transition energy and wavelength are given at the arrow)

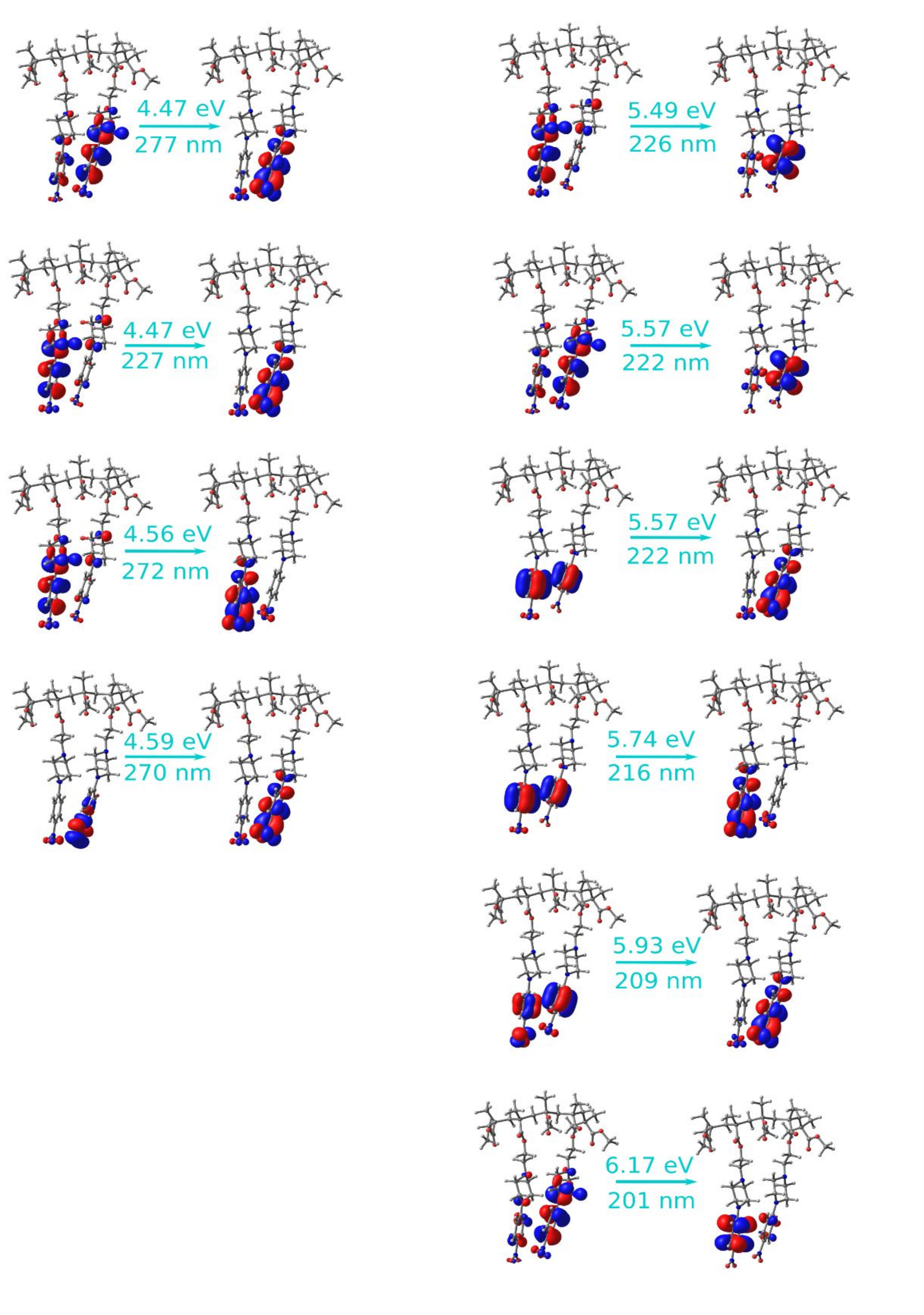

Figure S4. Frontier molecular orbitals of OK1 dimer

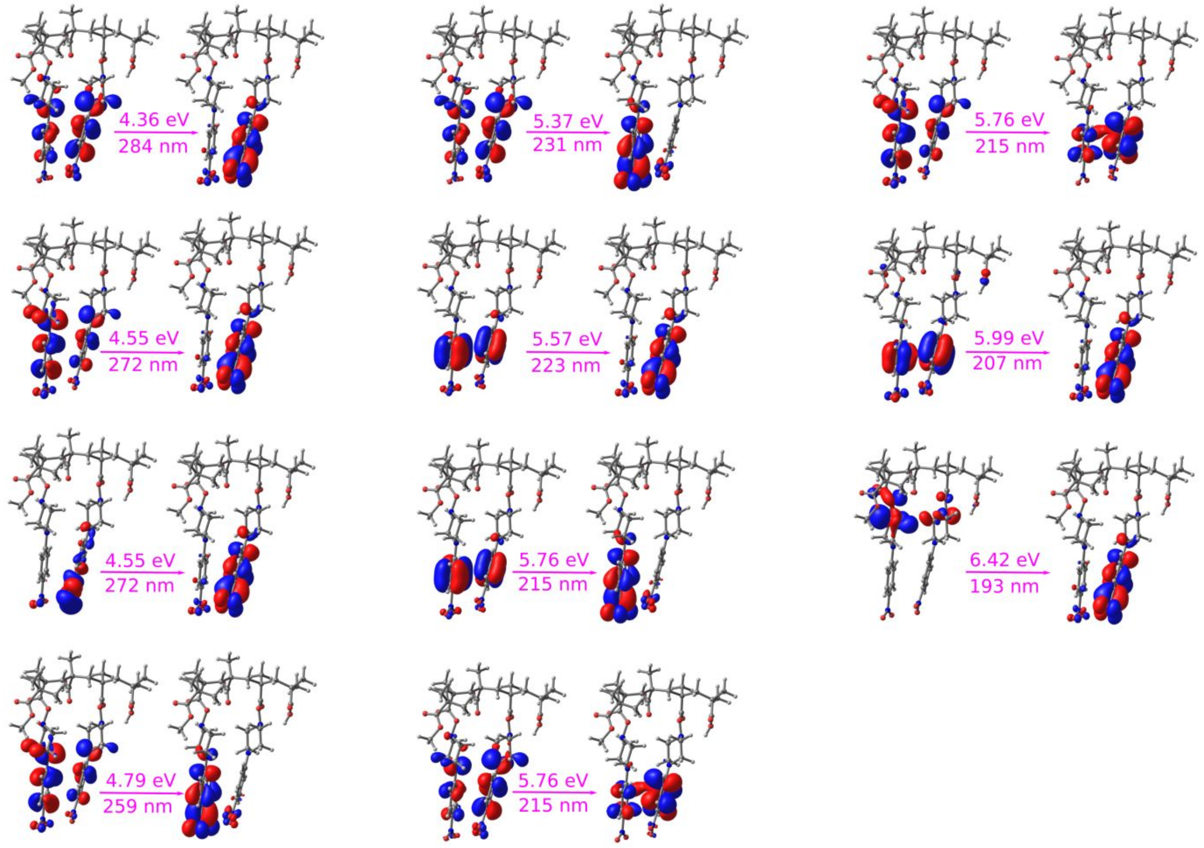

Figure S5. Frontier molecular orbitals of OK2 dimer

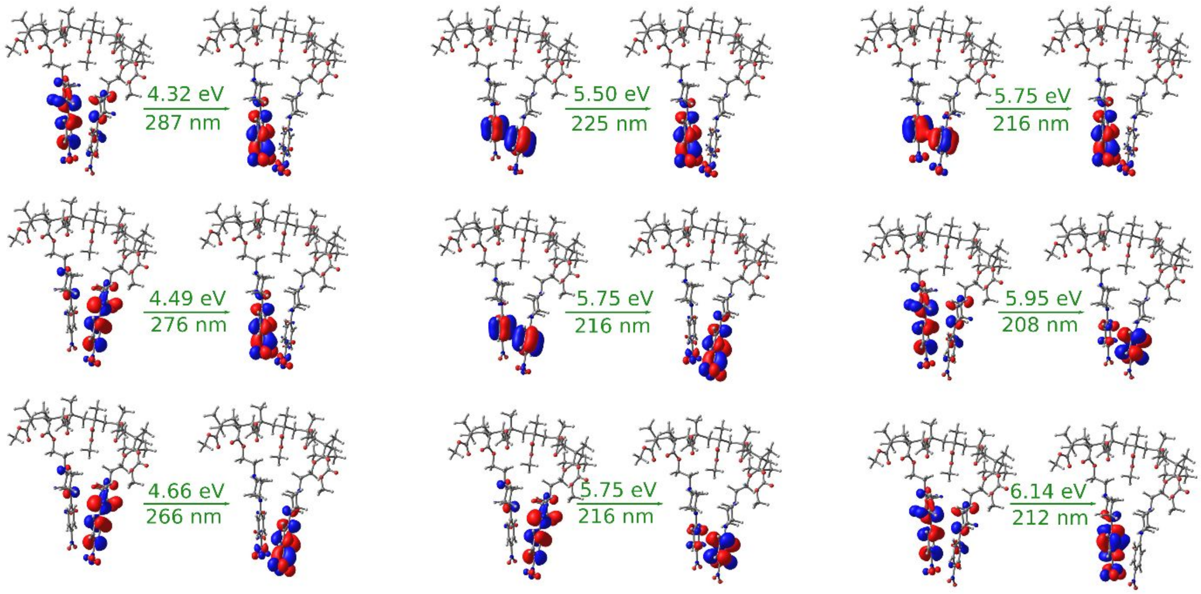

Figure S6. Frontier molecular orbitals of OK3 dimer

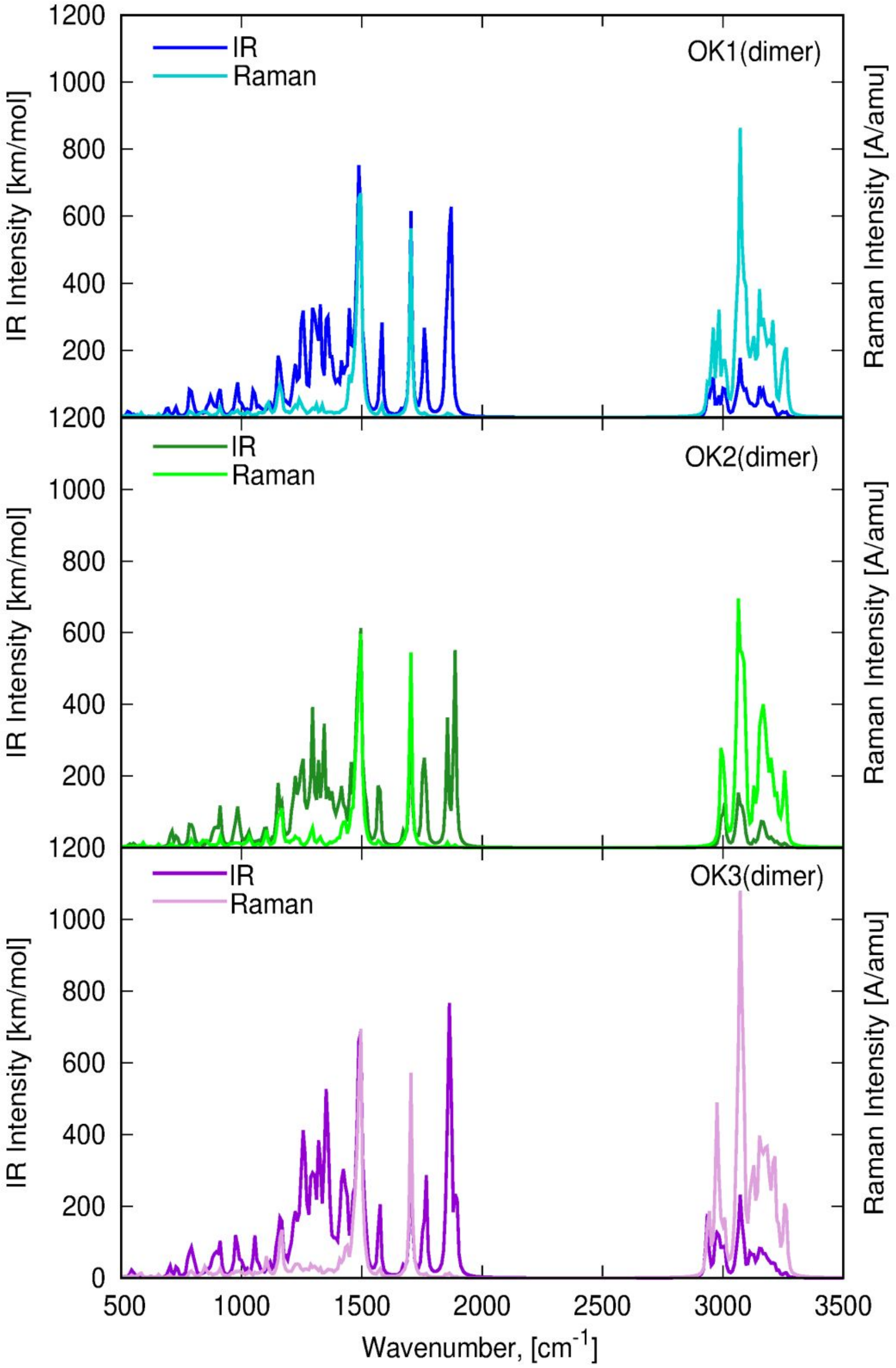

Figure S7. Theoretical IR and Raman spectra for investigated dimers

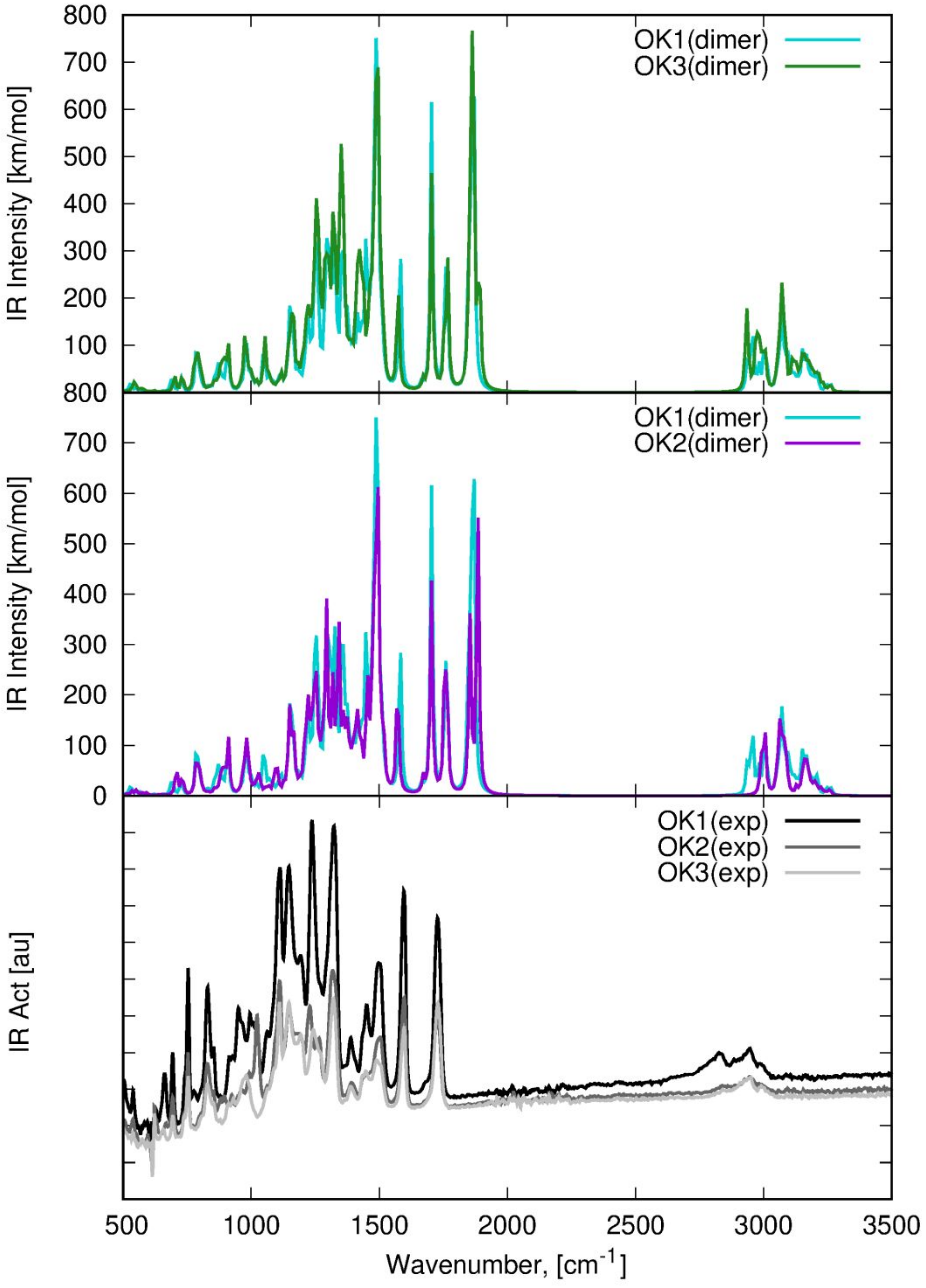

Figure S8. The ωB97X-D/def2-SVP IR spectrum for OK1, OK2 and OK3 dimers (upper panel: the influence of the linker length on the theoretical spectrum of the dimers, middle panel: the

influence of the distance between two chromophores on the theoretical spectrum of the dimers, lower panel for comparison: experimental FT-IR spectrum for OK1, OK2 and OK3 powders)

Table S1. Influence of the length of the linker in the polymer chain on the dye absorption spectrum

OK1/OK3- unit				OK1-dimer			
E (eV)	λ (nm)	f	orbs	E (eV)	λ (nm)	f	orbs
4.4415	279.15	0.5648	HOMO→LUMO	4.4699	277.38	0.2123	HOMO-1→LUMO<br>HOMO→LUMO
				4.5587	271.98	0.5875	HOMO-1→LUMO
				4.5923	269.98	0.1029	HOMO-16→LUMO
5.6742	218.50	0.0702	HOMO-3 →LUMO	5.4916	225.77	0.0218	HOMO→LUMO+2
				5.5730	222.47	0.0272	HOMO-1→LUMO+2<br>HOMO-4→LUMO

							HOMO→LUMO+2
				5.7455	215.79	0.0342	HOMO-4→LUMO+1
				5.9349	208.91	0.0257	HOMO-6→LUMO
				6.1734	200.84	0.0381	HOMO-1→LUMO+3
				OK3-dimer			
				4.3162	287.25	0.0673	HOMO-1→LUMO
				4.4884	276.23	0.4679	HOMO→LUMO
				4.6604	266.04	0.2989	HOMO→LUMO+1
				5.5006	225.40	0.0488	HOMO-4→LUMO
				5.7500	215.63	0.0220	HOMO-4→LUMO+1<br>HOMO→LUMO+3
				5.9522	208.30	0.0276	HOMO-9→LUMO
				6.1386	201.98	0.0321	HOMO-1→LUMO+3<br>HOMO-1→LUMO+7

Table S2. Influence of the distance from the polymer chain on the dye absorption spectrum

OK1/OK3- unit				OK1-dimer			
E (eV)	λ (nm)	f	orbs	E (eV)	λ (nm)	f	orbs
4.4415	279.15	0.5648	HOMO→LUMO	4.4699	277.38	0.2123	HOMO-1→LUMO<br>HOMO→LUMO
				4.5587	271.98	0.5875	HOMO-1→LUMO
				4.5923	269.98	0.1029	HOMO-16→LUMO
5.6742	218.50	0.0702	HOMO-3 →LUMO	5.4916	225.77	0.0218	HOMO→LUMO+2
				5.5730	222.47	0.0272	HOMO-1→LUMO+2<br>HOMO-4→LUMO<br>HOMO→LUMO+2
				5.7455	215.79	0.0342	HOMO-4→LUMO+1

				5.9349	208.91	0.0257	HOMO-6→LUMO
				6.1734	200.84	0.0381	HOMO-1→LUMO+3
OK2- unit				OK2-dimer			
4.4633	277.78	0.5670	HOMO→LUMO	4.3640	284.11	0.2031	HOMO→LUMO<br>HOMO-1→LUMO
5.6890	217.94	0.0716	HOMO-2→LUMO	4.5537	272.27	0.4504	HOMO→LUMO+1<br>HOMO-1→LUMO+1<br>HOMO→LUMO
				4.5804	270.69	0.0538	HOMO-18→LUMO
				4.7920	258.73	0.0568	HOMO→LUMO+2
				4.8887	253.61	0.0225	HOMO-1→LUMO
				5.3704	230.87	0.0276	HOMO-1→LUMO+1
				5.5667	222.73	0.0480	HOMO-4→LUMO
				5.7603	215.24	0.0460	HOMO-4→LUMO+1

				5.9867	207.10	0.0195	HOMO-9→LUMO+9
				6.3504	195.24	0.0439	HOMO-2→LUMO+4
				6.4203	193.11	0.0865	HOMO-2→LUMO+5
